# Case report: Unilateral transnasal endoscopic marsupialization of nasopalatine duct cyst

**DOI:** 10.3389/fsurg.2022.978915

**Published:** 2022-08-12

**Authors:** Ryoji Kagoya, Tomoko Iwanami, Makoto Mochizuki, Kenji Kondo, Ken Ito

**Affiliations:** ^1^ Department of Otolaryngology, Faculty of Medicine, Teikyo University, Tokyo, Japan; ^2^ Department of Otorhinolaryngology-Head and Neck Surgery, Graduate School of Medicine, The University of Tokyo, Tokyo, Japan; ^3^ Department of Clinical Laboratory Science, Faculty of Medical Technology, Teikyo University, Tokyo, Japan

**Keywords:** nasopalatine duct cyst, endoscopic marsupialization, nasopalatine nerve, oronasal fistula, paresthesia

## Abstract

Nasopalatine duct cyst (NPDC) is the most common type of non-odontogenic cysts of the jaw. It has been treated with complete surgical resection using a sublabial or palatine approach. However, complete removal of the cyst can be accompanied by postoperative complications including oronasal fistula. Recently, endoscopic marsupialization for the disease has been advocated, but there are still few reports regarding the surgery. Herein, we report a case of NPDC that was treated with unilateral transnasal endoscopic marsupialization. A 43-year-old man with no relevant previous medical history was referred to our hospital for the treatment of lesion occupying the right nasal cavity. A computerized tomography scan of the sinus revealed an egg-shaped lesion with a well-defined border centered on the lower half of the nasal cavity and hard palate. Based on the site of the lesion, it was considered to be NPDC. Transnasal endoscopic marsupialization was performed to diagnose and improve nasal obstruction. Histopathological examination revealed stratified squamous epithelium without atypia, which was consistent with NPDC. Although the patient noticed paresthesia of the right upper incisor area, symptoms improved 3 months after surgery. Written informed consent was obtained from the patient for the publication of any potentially identifiable images or data included in this article. Transnasal endoscopic marsupialization for NPDC is minimally invasive and useful; however, it is necessary to build evidence for an appropriate excision range based on the position and size of the lesion.

## Introduction

Nasopalatine duct cysts (NPDCs) are benign non-odontogenic lesions arising from epithelial remnants of the nasopalatine duct (1). It is the most common non-odontogenic cyst of the jaw, accounting for 1% to 11.6% of all jaw cysts (2). NPDC is approximately three times more frequent in males than in females (3), and is most common in middle-aged individual (4). To date, the standard treatment for NPDC has been complete surgical removal using a sublabial or palatine approach (1, 3, 5). However, complication such as oronasal fistula formation is a major concern associated with the complete removal of NPDC (6). Because NPDC is not a tumor, marsupialization of the nasal cavity is considered an effective treatment option. Nevertheless, few reports have detailed the method of endoscopic marsupialization for NPDC. Here, we report a case of NPDC that was treated with unilateral transnasal endoscopic marsupialization.

## Case presentation

**Figure 1:**
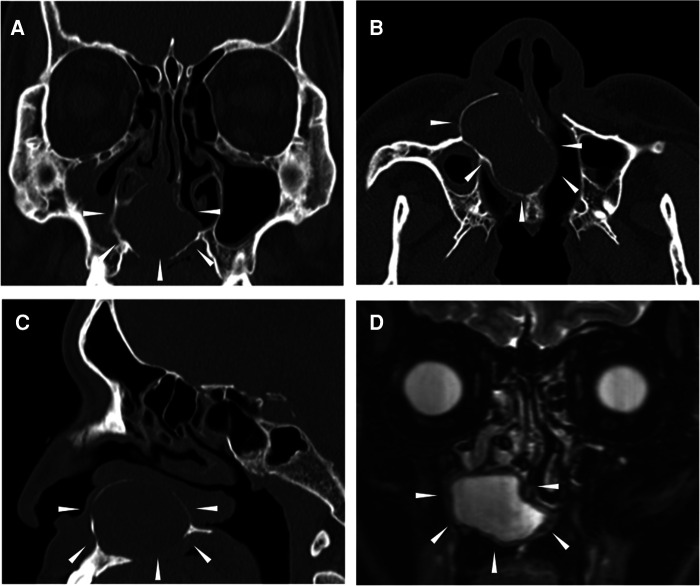
Preoperative CT scans and T2-weighted MRI of the sinuses. The area surrounded by the arrowhead indicates the lesion. (**A**) Coronal CT, (**B**) Axial CT, (**C**) Sagittal CT, and (**D**) T2-weighted MRI. CT, computed tomography; MRI, magnetic resonance image.

A 43-year-old man with a several-year history of nasal obstruction and discomfort was referred to our hospital from a primary care clinic. He had no relevant comorbidities or history of facial trauma. On nasal endoscopy, a smooth-surfaced mass excluding the inferior turbinate to the outside was observed in the right common nasal meatus. A computed tomography (CT) scan of the sinus revealed an egg-shaped lesion with a well-defined border centered on the lower half of the nasal cavity and hard palate, 32 mm × 28 mm × 36 mm in size (Figures 1A,C). The lesion was thought to originate from the nasopalatine duct. On T2-weighted magnetic resonance imaging (MRI), the lesion showed a uniform high intensity (Figure 1D). These findings were consistent with those of the NPDC.

**Figure 2:**
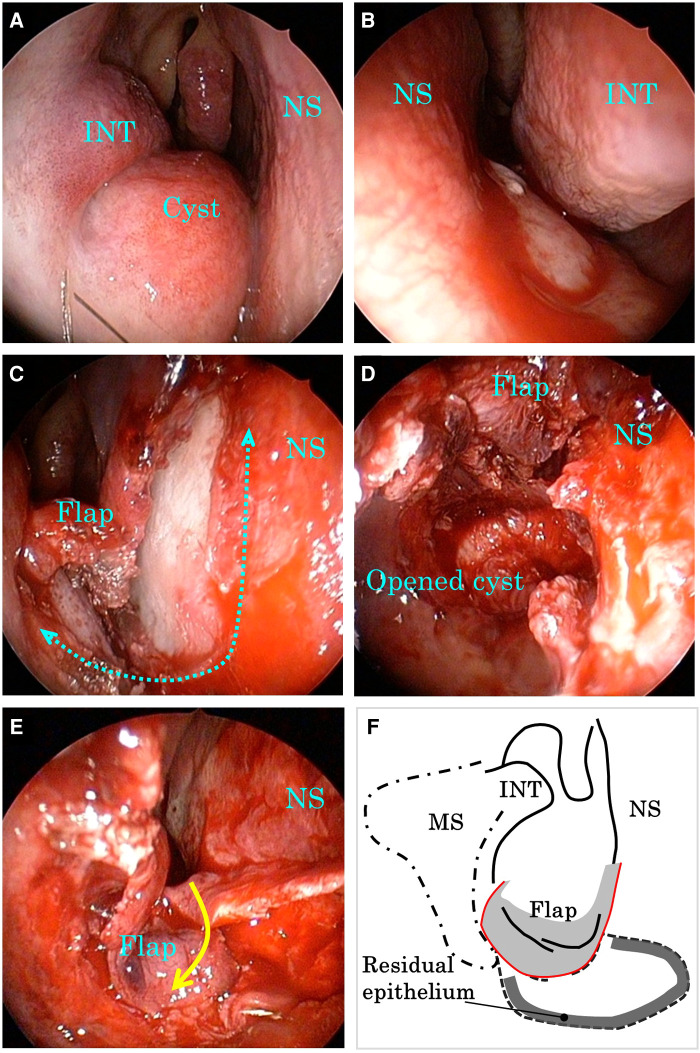
Intraoperative endoscopic images and schematic view. (**A**) Right nasal cavity at the beginning of the operation. (**B**) Left nasal cavity at the beginning of the operation. (**C**) Right nasal cavity after L-shaped mucosal incision. (**D**) Right nasal cavity after resection of the nasal part of the cyst. (**E**) Right nasal cavity after marsupialization of the cyst. (**F**) Schematic view of the surgery. INT, inferior nasal turbinate; MS, maxillary sinus; NS, nasal septum.

**Figure 3:**
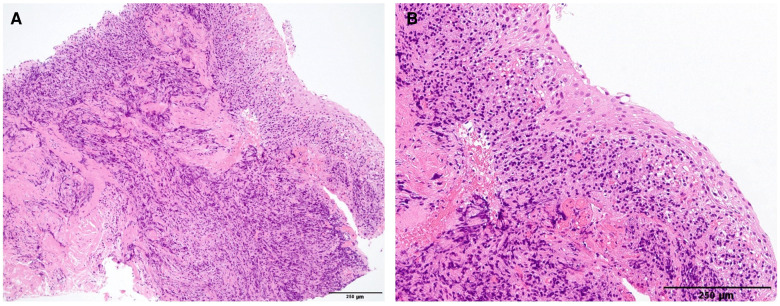
Hematoxylin and eosin-stained section of the resected cyst (scale bar = 250 μm). (**A, B**) Show weakly and highly magnified images, respectively. The cyst was lined by a stratified squamous epithelium with dense lymphocyte infiltration and fibrous tissue under the epithelium.

For improving his symptoms and diagnosis, transnasal endoscopic surgery was performed under general anesthesia. Large bulges from the nasal septum to the nasal floor were observed on the right side, and only slight bulges were observed on the left (Figures 2A,B). Therefore, marsupialization was only performed in the right nasal cavity. An L-shaped mucosal incision was made from the right side of the nasal septum to the nasal floor (Figure 2C). The mucoperiosteal flap was elevated and the bony cystic wall was exposed. The cyst wall was incised and the white cloudy cyst content was drained. Because the bony cystic wall was not very thick, it was cut with inferior turbinate scissors. Electrocautery equipment was required to detach the mucoperiosteal flap from the cyst. The cyst facing the nasal cavity was divided into several parts and resected, whereas the part facing the palate was preserved to avoid opening the nasal passages into the oral cavity. The anterolateral part of the cyst was also preserved considering the risk of infraorbital nerve damage. The exposed bone surface of the lateral wall of the nasal cavity was covered with a pedicled mucoperiosteal flap (Figures 2D,F). Histopathological examination revealed stratified squamous epithelium with dense lymphocyte infiltration, fibrous tissue under the epithelium, and no atypical cells (Figures 3A,B). These findings are consistent with those of the NPDC.

**Figure 4:**
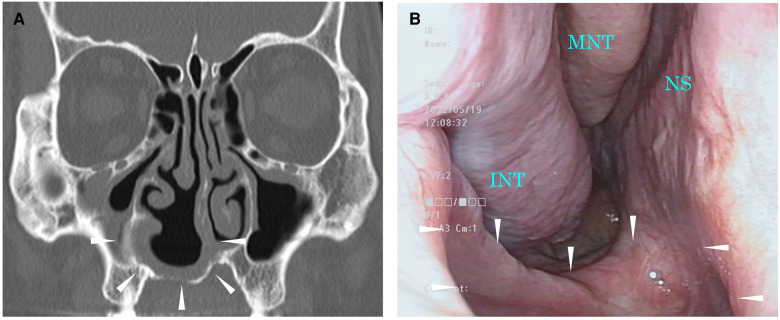
Images 3 months after surgery. The area surrounded by the arrowhead indicates a surgically opened area. (**A**) CT scan of the sinuses. (**B**) Endoscopic image of the right nasal cavity. CT, computed tomography; INT, inferior nasal turbinate; MNT, middle nasal turbinate; NS, nasal septum.

After surgery, nasal obstruction and discomfort disappeared. The patient noticed postoperative paresthesia of the right upper incisor area. The symptoms remitted 3 months after surgery. Paralysis of infraorbital nerve was not observed. Three months after surgery, the marsupialized cyst remained patent (Figures 4A,B). Written informed consent was obtained from the patient for the publication of any potentially identifiable images or data included in this article.

## Discussion

The symptoms of NPDC include swelling of the anterior palate and incisor discomfort. When it is large and is located in the upper part of the duct, nasal obstruction may occur (7). Pain and drainage can also occur if the cyst becomes infected. NPDCs are often asymptomatic and are discovered on routine physical examination or imaging for diagnosing other diseases (4).

Typical CT findings include well-defined round, oval, or heart-shaped lesions with a diameter of 1 cm–2 cm, located on or close to the midline of the upper maxilla (4). The cause of the heart shape is the presence of anterior nasal spines (4). The nasopalatine duct is a Y-shaped duct that opens into the incisive foramen of the palate and bifurcates to the left and right on the nasal side (8). Therefore, bilateral or unilateral occurrence also exists (9, 10), albeit most NPDCs are located in the midline of the maxilla. In the present case, it was presumed that the cyst originated from the area of the nasopalatine duct near the right nasal cavity. Histologically, NPDC is lined with stratified squamous epithelium alone or in combination with respiratory-type epithelium (11). A respiratory-type epithelium can be observed when the lesion is located near the nasal cavity (6).

There is a report of a case in which squamous cell carcinoma originated from NPDC in the anterior zone of the upper maxilla (12). Treatment for NPDC has traditionally been complete removal of the lesion by a transoral palatal or vestibular approach (6), considering the possibility of malignant tumor development. However, total resection is often difficult, particularly in cases of large NPDCs. In addition, there is a possibility of severe postoperative complications such as injury to the tooth roots, paresthesia of the anterior palate, and oronasal fistula formation (6). Recently, a few cases of NPDCs treated with transnasal endoscopic marsupialization have been reported (7, 13, 14). Marsupialization is a highly useful treatment because it can avoid deterioration of the quality of life due to oronasal fistula formation. Conversely, transient paresthesia of the upper incisor area occurred postoperatively in both the reported case (13) and our case. Given the origin of NPDCs, reliably preventing damage to the nasopalatine nerve may be difficult and remain a challenge in the future. Because there are variations in the location and size of NPDCs (9, 10), it is important to assess the risk of nerve damage before determining the extent of resection. In our case, the anterolateral part of the NPDC and the part on the palate was preserved, considering the risk of infraorbital nerve damage, because the lesion was extending widely to the right maxillary anterior wall. Consequently, buccal paralysis can be prevented. Running variations of the infraorbital nerve have been reported in cases of maxillary cysts (15). Thus, evaluation of the infraorbital nerve may also be important in cases of large NPDCs that extend anterolaterally. There is a limitation to the transnasal endoscopic marsupialization that should be recognized. It is difficult to observe and resect the anterior wall of the large cysts that extend anteriorly even with oblique-viewing endoscope. Bi-nostril transseptal approach may be useful when the infraorbital nerve is far away from the cyst and resection of the anterior wall is desirable.

## Conclusion

Here, we report a case of NPDC treated with unilateral transnasal endoscopic marsupialization. Endoscopic marsupialization may be an effective treatment option because it can reduce the risk of oronasal fistula formation. However, there are still few reports on the surgery, and a method to reliably prevent damage to the nasopalatine nerve has not been established. Careful follow-up and accumulation of a greater number of cases from multiple centers are necessary to build evidence for an appropriate excision range based on the position and size of the lesion.

## Data Availability

The original contributions presented in the study are included in the article/Supplementary Material, further inquiries can be directed to the corresponding author/s.

## References

[1] Swanson KS Kaugars GE Gunsolley JC. Nasopalatine duct cyst: An analysis of 334 cases. J Oral Maxillofac Surg. (1991) 49:268–71. 10.1016/0278-2391(91)90217-A 1995816

[2] Suter VG Sendi P Reichart PA Bornstein MM. The nasopalatine duct cyst: An analysis of the relation between clinical symptoms, cyst dimensions, and involvement of neighboring anatomical structures using cone beam computed tomography. J Oral Maxillofac Surg. (2011) 69(10):2595–603. 10.1016/j.joms.2010.11.032 21398010

[3] Escoda Francolí J Almendros Marqués N Berini Aytés L Gay Escoda C. Nasopalatine duct cyst: Report of 22 cases and review of the literature. Med Oral Patol Oral Cir Bucal. (2008) 13(7):E438–43. http://www.medicinaoral.com 18587308

[4] Falci SGM Verli FD Consolaro A dos Santos CRR. Morphological characterization of the nasopalatine region in human fetuses and its association to pathologies. J Appl Oral Sci. (2013) 21:250–5. 10.1590/1679-775720130008 23857659 PMC3881900

[5] Aldelaimi TN Khalil AA. Diagnosis and surgical management of nasopalatine duct cysts. J Craniofac Surg. (2012) 23:e472–4. 10.1097/SCS.0b013e318258764b 22976713

[6] Elliott KA Franzese CB Pitman KT. Diagnosis and surgical management of nasopalatine duct cysts. Laryngoscope. (2004) 114(8):1336–40. 10.1097/00005537-200408000-00004 15280704

[7] Wu PW Lee TJ Huang CC Huang CC. Transnasal endoscopic marsupialization for a huge nasopalatine duct cyst with nasal involvement. J Oral Maxillofac Surg. (2013) 71(5):891–3. 10.1016/j.joms.2012.11.002 23280191

[8] Jacobs R Lambrichts I Liang X Martens W Mraiwa N Adriaensens P Neurovascularization of the anterior jaw bones revisited using high-resolution magnetic resonance imaging. Oral Surg Oral Med Oral Pathol Oral Radiol Endod. (2007) 103(5):683–93. 10.1016/j.tripleo.2006.11.014 17320428

[9] Cicciù M Grossi GB Borgonovo A Santoro G Pallotti F Maiorana C. Rare bilateral nasopalatine duct cysts: Acase report. Open Dent J. (2010) 4:8–12. 10.2174/1874210601004010008 20386720 PMC2852121

[10] Wu YH Wang YP Kok SH Chang JY. Unilateral nasopalatine duct cyst. J Formos Med Assoc. (2015) 114(11):1142–4. 10.1016/j.jfma.2015.08.005 26329385

[11] Vasconcelos R de Aguiar MF Castro W de Araujo VC Mesquita R. Retrospective analysis of 31 cases of nasopalatine duct cyst. Oral Dis. (1999) 5(4):325–8. 10.1111/j.1601-0825.1999.tb00098.x 10561722

[12] Takagi R Ohashi Y Suzuki M. Squamous cell carcinoma in the maxilla probably originating from a nasopalatine duct cyst: Report of case. J Oral Maxillofac Surg. (1996) 54(1):112–5. 10.1016/s0278-2391(96)90318-3 8530989

[13] Honkura Y Nomura K Oshima H Takata Y Hidaka H Katori Y. Bilateral endoscopic endonasal marsupialization of nasopalatine duct cyst. Clin Pract. (2015) 5(1):748; eCollection 2015 Jan 28. 10.4081/cp.2015.748 25918636 PMC4387348

[14] Kang JW Kim HJ Nam W Kim CH. Endoscopic endonasal marsupialization of nasopalatine duct cyst. J Craniofac Surg. (2014) 25(2):e155–6. 10.1097/SCS.0000000000000417 24448528

[15] Kondo K Baba S Suzuki S Nishijima H Kikuta S Yamasoba T. Infraorbital nerve located medially to postoperative maxillary cysts: A risk of endonasal surgery. ORL J Otorhinolaryngol Relat Spec. (2018) 80(1):28–35. 10.1159/000486372 29462811

